# Diagnostic and Prognostic Role of Serum Omentin and NGAL Levels in Egyptian Breast Cancer Patients

**DOI:** 10.1155/2022/5971981

**Published:** 2022-09-14

**Authors:** Al-Shimaa Mahmoud Abas, Mohamed H. Sherif, Sara A. Elmoneam Farag

**Affiliations:** ^1^Biochemistry Division, Chemistry Department, Faculty of Science, Zagazig University, Egypt; ^2^Chemistry Department, Faculty of Science, Zagazig University, Zagazig, Egypt

## Abstract

**Background:**

Breast cancer (BC) is globally the main cause of cancer-related deaths in women. Tumor biomarkers have significant role in diagnosis and predicting the prognosis and decide the specific therapy to each patient.

**Aim:**

In this study, we investigated whether omentin and NGAL levels were altered in patients with breast cancer and the relationship between these markers and their clinicopathological parameters. *Subjects and Methods*. This study included 120 patients with breast cancer and 30 healthy individuals served as controls. We measured the serum level of omentin and NGAL by ELISA technique.

**Results:**

Our results showed that there were statistically significant differences in serum omentin and NGAL levels between two groups. Also, in breast cancer patients, there was significant difference between omentin level, the same results with NGAL level and patient's age, tumor size, lymph node, and metastasis. No significant relationship was found between omentin level and tumor grade, ER, PR, and HER2. The cutoff value for the prediction of breast cancer was determined at >113.2 ng/ml for omentin and >145.3 ng/ml for NGAL with a sensitivity of 91.7% and 100%, specificity of 100% and 80%, positive predictive value of 100% and 90.9%, negative predictive value of 85.7% and 100%, and accuracy of 94.4% and 93.3%, respectively. In conclusion, serum omentin and NGAL can be used as strong diagnostic markers for breast cancer.

## 1. Introduction

The leading cause of cancer-related mortality in women globally is breast cancer (BC), one of the most frequent malignancies [1]. In response to cancer or some benign situations, tumors or other body cells create tumor markers [2].

Biomarkers could be prognostic, predictive, or both. Biomarkers measure prognosis independent of other factors. The presence or absence of these markers is directly related to recurrence or mortality of the disease. Markers can predict if a patient will respond to a certain therapy or not [3].

For many years, factors such as tumor size, axillary lymph node status, tumor histology, estrogen receptor (ER), progesterone receptor (PR), human epidermal growth factor receptor 2 (HER2), patient age, and prognosis were used to assess the prognosis and choose the best course of action for breast cancer patients [4].

Adipose tissue makes up the breast tissue. Therefore, adipose tissue's function in the development and spread of breast cancer was highlighted in a number of research. Leptin, chemerin, resistin, and omentin-1 are only a few of the adipokines that the adipose tissue makes and releases [5].

Omentin-1, a peptide with a molecular weight of 34 kDa, is an adipokine that is generated in visceral adipose tissue and has anti-inflammatory and anti-insulin resistance properties [6].

NGAL (lipocalin 2) is a protein found in human neutrophil, included in bone marrow during the maturation of granulocyte. It was included in different physiological and pathological conditions. Few number of studies were found to be related to BC [7].

The current study's objectives were to analyze the relationship between the blood levels of NGAL and omentin as prognostic markers and the potential utility of these markers for the detection of breast cancer.

## 2. Subjects and Methods

This study included 30 healthy persons and 120 patients with breast cancer admitted to Clinical Oncology and Internal Medicine Outpatient Clinics of Zagazig University Hospital. The data of this analytical descriptive study were obtained from patient's documents. The presence of estrogen receptor (ER), progesterone receptor (PR), human epidermal growth factor receptor-2 (HER2), lymph node (LN) status, tumor grades, and sizes were obtained after diagnosis. The grade of tumors was confirmed by a pathologist, and the lymph node status was confirmed clinically using imaging techniques after surgery. The criteria for selecting the patients were (a) presence of breast lump which was diagnosed as breast carcinoma; (b) no systemic disease such as diabetes mellitus, hypertension, chronic inflammatory disease, and liver, renal, or heart failure. The data recorded are partly presented in Table 1.

All volunteers signed consent for their participation in this study. Moreover, an approval was taken from the ethical committee in Zagazig University under the ethical consideration of Helsinki Declaration 1964.

### 2.1. Blood Sampling

All subjects had their venous blood samples taken while fasting. After a minimum of 30 minutes, the sera were separated by centrifugation at 3000 rpm for 10 minutes, and the remaining serum was collected, divided into aliquots, and kept at -80°C pending further processing.

### 2.2. Measurement of Circulating Human Omentin-1 and NGAL Level

Serum concentration of omentin and NGAL was measured using a commercially available ELISA kit purchased from Abcam (ab269545 and ab113326, respectively).

### 2.3. Statistical Analysis

Data were analyzed in SPSS 16.0. The analysis of variance (ANOVA) evaluated the relationship between the omentin-1 and NGAL with clinicopathological factors. A probability *p* value < 0.05 was considered statistically significant. The data were reported as means ± standard deviation.

## 3. Results

### 3.1. Baseline Clinical and Pathological Data

A total of 120 newly diagnosed breast cancer patients were enrolled in this study. The characteristics of these patients are shown in Table 1.

### 3.2. Relationship between Serum Level of Omentin-1 and Clinicopathological Characteristic of Breast Cancer Cases

Our results presented in Table 2 showed significant difference between omentin-1 level and patient's age (*p* < 0.05), tumor size (*p* < 0.001), lymph node (*p* < 0.05), and metastasis (*p* < 0.001). No significant relationship was found between omentin-1 level and tumor grade, ER, PR, and HER2 (*p* > 0.05).

### 3.3. Relationship between Serum Level of NGAL and Clinicopathological Characteristic of Breast Cancer Cases

Our results presented in Table 3 showed significant difference between NGAL level and patient's age (*p* < 0.05), tumor size (*p* < 0.001), lymph node (*p* < 0.01), and metastasis (*p* < 0.001). No significant relationship was found between NGAL level and tumor grade, ER, PR, and HER2 (*p* > 0.05).

### 3.4. Serum Level of Omenti-1 and NGAL in Healthy Control and Breast Cancer Patients

**Figure 1:**
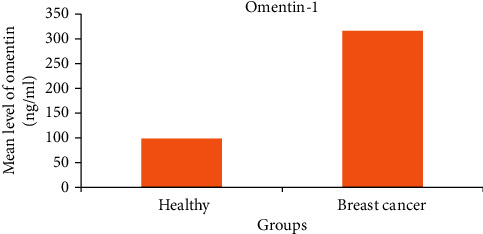
Mean level omentin-1 (ng/ml) in all studied groups.

**Figure 2:**
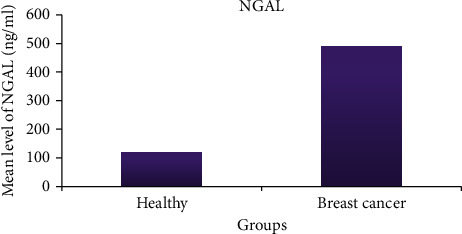
Mean level NGAL (ng/ml) in all studied groups.

Results in Table 4 documented that there was significant difference between the mean levels of Omenti-1 (98.4 ± 8.2 ng/ml) and NGAL (117.6 ± 15.7 ng/ml) in healthy control and breast cancer patients (316.5 ± 293.6 ng/ml and 488.5 ± 388 ng/ml), respectively (*p* < 0.001) (Figures 1 and 2).

### 3.5. Correlation Analysis between All the Parameters under Study

Our data showed that there was no correlation between Omenti-1 and age, tumor grade, ER, PR, and HER2. Also, positive relationship was found between Omenti-1 and tumor size (*r* = 0.480), lymph node (*r* = 0.425), and metastasis (*r* = 0.780) and strong positive relationship with NGAL (*r* = 0.805) (Table 5).

Also, our data showed that there was no correlation between NGAL and age, tumor grade, ER, PR, and HER2. Also, positive relationship was found between NGAL and tumor size (*r* = 0.401), lymph node (*r* = 0.331), and metastasis (*r* = 0.653).

### 3.6. Defining Cutoff Points for Serum Level of Omenti-1 and NGAL

**Figure 3:**
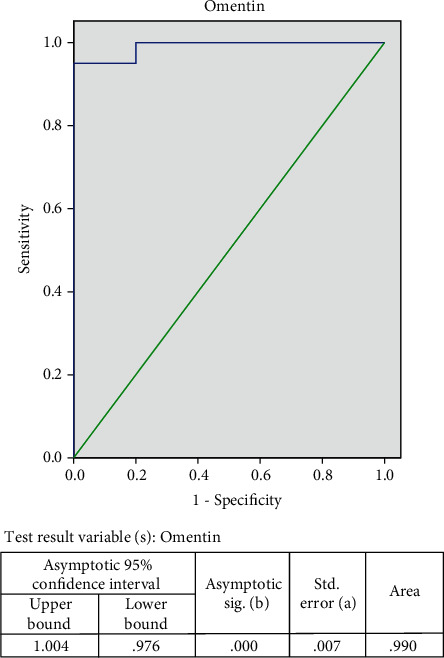
ROC curve for serum omentin-1 levels in the diagnosis of breast cancer.

The cutoff value of Omenti-1 and NGAL levels for predicting breast cancer was determined by ROC analysis. The cutoff value for the prediction of breast cancer was determined at >113.2 ng/ml for Omenti-1 with a sensitivity of 91.7%, specificity of 100%, positive predictive value of 100%, negative predictive value of 85.7%, and accuracy of 94.4% (AUC, 0.990) (Figure 3).

**Figure 4:**
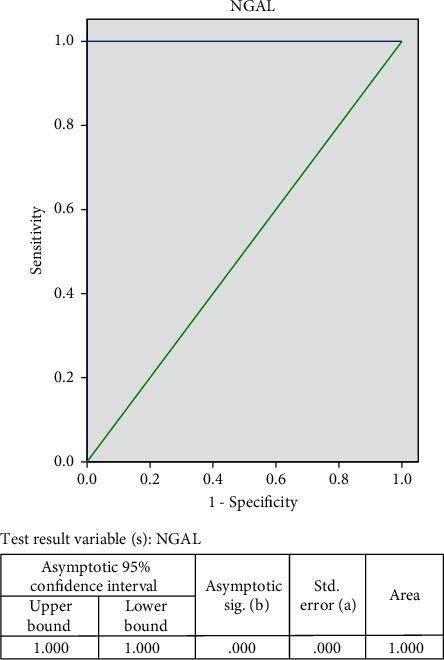
ROC curve for serum NGAL levels in the diagnosis of breast cancer.

Also, the cutoff value for the prediction of breast cancer was determined at >145.3 ng/ml for NGAL with a sensitivity of 100%, specificity of 80%, positive predictive value of 90.9%, negative predictive value of 100%, and accuracy of 93.3% (AUC, 0.100) (Figure 4).

## 4. Discussion

Breast cancer is highly variable in its etiology and pathology, with some patients having slow growth and excellent prognosis, while others had a very aggressive clinical course [8].

The stage of diagnosis and biological characteristics of the tumor have an impact on the likelihood of recurrence and prognosis. A major goal of breast cancer diagnosis is to improve the accuracy of biomarkers for early detection of disease.

Prognostic markers such as tumor size, grade, age, histology, and estrogen receptor status influence treatment decisions. This study was conducted to evaluate omentin-1 and NGAL to determine their prognostic and diagnostic value in breast cancer patients. In addition, we attempted to show the relationship between omentin-1, NGAL, and prognostic factors and their impact on disease outcome.

Our results presented in Table 2 showed significant increase in omentin-1 level and patients with age ≥ 40 years than patients with age < 40 years (*p* < 0.05). Also, there was a significant difference between tumor size (*p* < 0.001), lymph node (*p* < 0.05), and metastasis (*p* < 0.001). No significant difference was found between omentin-1 level and tumor grade, ER, PR, and HER2 (*p* > 0.05).

Our results are consistent with previous studies that found a positive association between omentin-1 and age (*r* = 0.26, *p* < 0.001) [9]. Serum omentin levels were significantly higher in patients with larger than small pathological tumor sizes (*p* = 0.03) [10].

Our results presented in Table 3 showed significant difference between NGAL level and patient's age (*p* < 0.05), tumor size (*p* < 0.001), lymph node (*p* < 0.01), and metastasis (*p* < 0.001). No significant relationship was found between HE4 level and tumor grade, ER, PR, and HER2 (*p* > 0.05).

In previous studies, estrogen receptor (ER) status (positive: negative), HER-2 receptor status (positive: negative), tumor size (≤1:>1), and lymph node metastasis (N0+ N1: N2+N3), and plasma NGAL levels (all *p* >0.05) [11].

Our results show the mean concentrations of omentin-1 (98.4 ± 8.2 ng/ml) and NGAL (117.6 ± 15 ng/ml) in healthy controls and breast cancer patients (316.5 ± 293.6 ng/ml). We have demonstrated that there is a significant difference (milliliters) (488.5 ± 388 ng/ml,*p* < 0.001).

This is the same result that found that omentin-1 is elevated in patients with both benign and malignant breast disease [9, 10]. Serum omentin-1 levels are elevated in patients with disease burden. This could be explained by activation of the Akt signaling pathway and/or inflammatory processes [12].

In cancer research, omentin induces cancer cell proliferation by activating genomic instability and the PI3K/Akt (phosphatidylinositol-3-kinase downstream effector) signaling pathway, suggesting that omentin's cancer-promoting effects were independent of its ability to reduce metabolic risk from obesity [13].

Also, other studies said that plasma NGAL levels were elevated significantly in patients with breast cancer [13].

NGAL is expressed in several other cell types in addition to neutrophils. If NGAL is found in complex with her MMP-9, the effect of NGAL on MMP-9 stability results in less MMP-9 degradation. This increases the gelatinolytic activity of MMP-9 in the extracellular matrix. NGAL can affect the development of various types of cancer [14]. NGAL is an acute-phase protein involved in various physiological processes in mice, including ion transport, apoptosis, inflammation, cell survival, and carcinogenesis [15].

Our data showed that there was no correlation between Omenti-1 and age, tumor grade, ER, PR, and HER2. Also, positive relationship was found between Omenti-1 and tumor size (*r* = 0.480), lymph node (*r* = 0.425), and metastasis (*r* = 0.780) and strong positive relationship with NGAL (*r* = 0.805).

Also, our data showed that there was no correlation between NGAL and age, tumor grade, ER, PR, and HER2. Also, positive relationship was found between NGAL and tumor size (*r* = 0.401), lymph node (*r* = 0.331), and metastasis (*r* = 0.653).

Our results show that the cutoff values for predicting breast cancer are >113.2 ng/ml for Omenti-1 with a sensitivity of 91.7%, a specificity of 100%, a positive predictive value of 100%, a negative predictive value of 85.7%, and accuracy of 94.4% (AUC, 0.990).

Another study revealed that the best cutoff point for the diagnosis of BC in the serum levels was at 136.5 ng/l. The sensitivity and specificity for the serum levels of omentin-1 with 95% CI (0.791 to 0.902) were 63.64% and 89.02%, respectively [16].

Also, our results showed that the cutoff value for the prediction of breast cancer was determined at >145.3 ng/ml for NGAL with a sensitivity of 100%, specificity of 80%, positive predictive value of 90.9%, negative predictive value of 100%, and accuracy of 93.3% (AUC, 0.100).

In another study, the serum NGAL cutoff value to differentiate healthy controls from breast cancer patients was 277.9 ng/ml, and the calculated sensitivity and specificity were 83% and 100%, respectively [17].

Our results suggested that omentin-1 and NGAL may be beneficial and invasive tumor markers in the diagnosis of patients with breast cancer.

## 5. Conclusion

The results of this study suggest that omentin-1 and NGAL serum levels are considered valuable potential biomarkers and can add to the prognostic information available from classical prognostic factors such as pathological tumor size, lymph node metastasis, and metastasis. This supports the finding. Furthermore, there is no significant association between omentin-1, NGAL, and prognostic factors such as hormone receptors and HER2. Finally, serum omentin-1 and NGAL can be used as strong diagnostic markers for breast cancer.

## Figures and Tables

**Table 1:** Clinicopathological data of analyzed cases.

		Number of cases	%
Age	<40	12	10
	≥40	108	90

Tumor size	T0-T1	24	20
	T2	24	20
	T3	24	20
	T4	48	40

Lymph node	N0	8	6.7
	N1	48	40
	N2	14	11.7
	N3	46	38.3
	Unknown (X)	4	3.3

Metastasis	Yes	24	20
	No	96	80

Tumor grade	G1	2	1.7
	G2	86	71.7
	G3	12	10
	Unknown (X)	20	16.6

Estrogen receptor (ER)	Positive	52	43.3
	Negative	48	40
	Unknown (X)	20	16.7

Progesterone receptor (PR)	Positive	40	33.3
	Negative	60	50
	Unknown (X)	20	16.7

HER2	Positive	14	11.7
	Negative	48	40
	Unknown (X)	58	48.3

**Table 2:** Relation between serum level of omentin-1 (ng/ml) and clinicopathological characteristic of the 120 studied cases.

		Omentin (ng/ml) (mean ± SD)	*p* value
Age	<40	179.7 ± 49.2	0.002
	≥40	333.9 ± 30.8	

Tumor size	T0-T1	161 ± 49.6	<0.001
	T2	185.9 ± 41.8	
	T3	230.3 ± 43.6	
	T4	501.2 ± 397.5	

Lymph node	N0	140.5 ± 20.4	0.001
	N1	198.8 ± 47.2	
	N2	224.5 ± 38.1	
	N3	514.1 ± 401.4	
	Unknown (X)	133.2 ± 37.7	

Metastasis	Yes	770.5 ± 412.9	0.001
	No	203.1 ± 50.5	

Tumor grade	G1	275.1 ± 32.8	0.41
	G2	301.4 ± 279.6	
	G3	212.3 ± 35.8	
	Unknown (X)	448.4 ± 414.5	

Estrogen receptor (ER)	Positive	406.5 ± 381.9	0.08
	Negative	274.3 ± 208.2	
	Unknown (X)	184 ± 40.9	

Progesterone receptor (PR)	Positive	463.8 ± 420	0.15
	Negative	262.6 ± 187.9	
	Unknown (X)	184 ± 40.9	
HER2neu	Positive	283.5 ± 288.5	0.85
	Negative	341.9 ± 321.7	
	Unknown (X)	303.6 ± 278.9	

**Table 3:** Relation between serum level of NGAL (ng/ml) and clinicopathological characteristic of the 120 studied cases.

		NGAL (ng/ml) (mean ± SD)	*p* value
Age	<40	331.5 ± 72.8	0.006
	≥40	509.3 ± 408	

Tumor size	T0-T1	332.1 ± 60.6	0.005
	T2	325.7 ± 43.6	
	T3	383.3 ± 62.6	
	T4	700.8 ± 549.7	

Lymph node	N0	342.6 ± 41	0.03
	N1	359.4 ± 78.5	
	N2	400.5 ± 46.4	
	N3	687.2 ± 573.7	
	Unknown (X)	353.6 ± 0	

Metastasis	Yes	990.9 ± 665.3	0.007
	No	362.9 ± 69.7	

Tumor grade	G1	466.1 ± 62.1	0.23
	G2	445.1 ± 334.1	
	G3	417.5 ± 62.0	
	Unknown (X)	720.4 ± 625.9	

Estrogen receptor (ER)	Positive	530.1 ± 449.8	0.58
	Negative	488.9 ± 397.3	
	Unknown (X)	379.9 ± 57.8	

Progesterone receptor (PR)	Positive	562.4 ± 510	0.46
	Negative	475.6 ± 355.6	
	Unknown (X)	379.6 ± 57.8	

HER2neu	Positive	544.5 ± 539.8	0.83
	Negative	507.2 ± 458.4	
	Unknown (X)	459.6 ± 283.6	

**Table 4:** Serum omentin (ng/ml) and NGAL (ng/ml) in different studied groups.

Markers		Study groups	
		Healthy (*N* = 30)	Breast cancer patients (*N* = 120)
Omentin-1 (ng/ml)	Mean ± SD	98.4 ± 8.2	316.5 ± 293.6
	Minimum	91.7	106.5
	Maximum	113.2	1172.7
	*p* value	<0.001	

NGAL (ng/ml)	Mean ± SD	117.6 ± 15.7	488.5 ± 388
	Minimum	99.5	217.8
	Maximum	145.3	1917
	*p* value	<0.001	

**Table 5:** Correlation analysis between omentin-1 and NGAL and clinicopathological data of all cases under study.

Parameter	Spearman rho^∗^	Omentin-1	NGAL
Age	*p*	Insignificant (*p* > 0.05)	Insignificant (*p* > 0.05)
	*r*		
Tumor size	*p*	Significant (*p* < 0.001)	Significant (*p* = 0.001)
	*r*	0.480	0.401
Lymph node	*p*	Significant (*p* = 0.001)	Significant (*p* = 0.01)
	*r*	0.425	0.331
Metastasis	*p*	Significant (*p* < 0.001)	Significant (*p* < 0.001)
	*r*	0.780	0.653
Tumor grade	*p*	Insignificant (*p* > 0.05)	Insignificant (*p* > 0.05)
	*r*		
Estrogen receptor (ER)	*p*	Insignificant (*p* > 0.05)	Insignificant (*p* > 0.05)
	*r*		
Progesterone receptor (PR)	*p*	Insignificant (*p* > 0.05)	Insignificant (*p* > 0.05)
	*r*		
HER2neu	*p*	Insignificant (*p* > 0.05)	Insignificant (*p* > 0.05)
	*r*		
	*r*	0.664	0.646
Omentin-1	*p*	—	Significant (*p* < 0.001)
	*r*		0.805
NGAL	*p*	Significant (*p* < 0.001)	—
	*r*	0.805	

## Data Availability

The authors confirm that the data supporting the findings of this study are available within the article.

## References

[1] Bray F., Ferlay J., Soerjomataram I., Siegel R. L., Torre L. A., Jemal A. (2018). Global cancer statistics 2018: GLOBOCAN estimates of incidence and mortality worldwide for 36 cancers in 185 countries. *CA: a Cancer Journal for Clinicians*.

[2] Geng B., Liang M. M., Ye X. B., Zhao W. Y. (2015). Association of CA 15-3 and CEA with clinicopathological parameters in patients with metastatic breast cancer. *Molecular and Clinical Oncology*.

[3] Taneja P., Maglic D., Kai F. (2010). Classical and novel prognostic markers for breast cancer and their clinical significance. *Clinical Medicine Insights: Oncology*.

[4] Kittaneh M., Montero A. J., Glück S. (2013). Molecular pro ling for breast cancer: a comprehensive review. *Biomark. Cancer*.

[5] Bougaret L., Delort L., Billard H. (2018). Adipocyte/breast cancer cell crosstalk in obesity interferes with the anti-proliferative efficacy of tamoxifen. *PLoS One*.

[6] Li Z., Liu B., Zhao D. (2017). Omentin-1 prevents cartilage matrix destruction by regulating matrix metalloproteinases. *Biomedicine & Pharmacotherapy*.

[7] Wang Y. U., Zeng T. (2013). Neutrophil gelatinase-associated lipocalin protein as a biomarker in the diagnosis of breast cancer: a meta-analysis. *Biomedical. Reports*.

[8] Abdel-Aziz T., Azab N., Emara N., Odah M., El-deen I. M. (2015). Study of BRCA2 gene mutations in Egyptian females with breast cancer. *Breast Cancer*.

[9] Panagiotou G., Triantafyllidou S., Tarlatzis B. C., Papakonstantinou E. (2021). Serum levels of irisin and omentin-1 in breast neoplasms and their association with tumor histology. *International Journal of Endocrinology*.

[10] Karabulut S., Afsar C. U., Karabulut M. (2016). Clinical significance of serum omentin-1 levels in patients with pancreatic adenocarcinoma. *BBA Clinical*.

[11] Wei C. T., Tsai I. T., Wu C. C. (2021). Elevated plasma level of neutrophil gelatinase-associated lipocalin (NGAL) in patients with breast cancer. *International Journal of Medical Sciences*.

[12] Aleksandrova K., di Giuseppe R., Isermann B. (2016). Circulating omentin as a novel biomarker for colorectal cancer risk: data from the EPIC-potsdam cohort study. *Cancer. Research*.

[13] Wu S. S., Liang Q. H., Liu Y., Cui R. R., Yuan L., Liao E. Y. (2013). Omentin-1 stimulates human osteoblast proliferation through PI3K/Akt signal pathway. *International Journal of Endocrinology*.

[14] Candido S., Maestro R., Polesel J. (2014). Roles of neutrophil gelatinase-associated lipocalin (NGAL) in human cancer. *Oncotarget*.

[15] Chakraborty S., Kaur S., Guha S., Batra S. K. (2012). The multifaceted roles of neutrophil gelatinase associated lipocalin (NGAL) in inflammation and cancer. *Biochimica et Biophysica Acta*.

[16] Tahmasebpour N., Hosseinpour M. A. H., Ziamajidi N. (2020). Association of omentin-1 with oxidative stress and clinical significances in patients with breast cancer. *Advanced Pharmaceutical Bulletin*.

[17] Mohammed F. Z., Gamal L., Mosa M. F., El-deen I. M. (2020). Study of the potential role of serum neutrophil gelatinase-associated lipocalin (NGAL) levels in the diagnosis and prognosis of breast cancer in Egyptian females a case-control study. *Asian Journal of Research in Biochemistry*.

